# Noncoding SNPs decrease expression of FABP5 during COPD exacerbations

**DOI:** 10.1172/JCI175626

**Published:** 2024-02-01

**Authors:** Manale El Kharbili, Sarah K. Sasse, Lynn Sanford, Sean Jacobson, Katja Aviszus, Arnav Gupta, Claire Guo, Susan M. Majka, Robin D. Dowell, Anthony N. Gerber, Russell P. Bowler, Fabienne Gally

**Affiliations:** 1 Department of Immunology and Genomic Medicine and; 2 Department of Medicine, National Jewish Health, Denver, Colorado, USA.; 3 BioFrontiers Institute,; 4 Department of Medicine,; 5 Molecular, Cellular and Developmental Biology, and; 6 Computer Science, University of Colorado, Boulder, Colorado, USA.

**Keywords:** Genetics, Metabolism, COPD

**To the Editor:** The pathogenesis of chronic obstructive pulmonary disease (COPD) is characterized by abnormal airway inflammation progressing to airflow obstruction and tissue destruction (1). While cigarette smoking and genetics clearly contribute to COPD risk, infections and associated exacerbations are also linked to disease pathogenesis (2). It is thought that systemic inflammation in COPD is a consequence of “spillover” of inflammatory mediators from the lungs. However, the mechanisms leading to the amplification of inflammation during episodes of exacerbation are largely unidentified.

Fatty acid binding protein 5 (FABP5) is a small cytoplasmic protein involved in fatty acid transport and metabolism. We previously reported that FABP5 is downregulated in COPD and further downregulated in patients reporting one or more exacerbations (3). Here, we investigated the potential impact of genetic variation in *FABP5*. We performed a negative binomial analysis on the COPDGene SNP data set (https://www.copdgene.org/) and identified 5 linked single-nucleotide polymorphisms (SNPs) within the *FABP5* locus that are significantly associated with severe exacerbations in a non-Hispanic White cohort (Figure 1A). To date, none of these SNPs (rs4338057, rs12549270, rs202275, rs202277, and rs202279) have been characterized to the best of our knowledge (Supplemental Table 1; supplemental material available online with this article; https://doi.org/10.1172/JCI175626DS1). The relative DNase sensitivity, enrichment for regulatory histone modifications, and overrepresentation of ENCODE-defined transcription factor binding sites in the rs4338057, rs202275, and rs202277 SNP regions implicate these regions in conferring regulatory activity (Figure 1B). To probe this regulatory potential further, we first visualized our assay for transposase-accessible chromatin with sequencing (ATAC-seq) data in patient-derived airway epithelial cells (4) across the *FABP5* locus, and observed a robust peak of accessible chromatin overlapping the rs202275 region (Figure 1C). We then aligned our recent precision run-on sequencing (PRO-seq) data set (5) and found the rs202275 region marked by a bidirectional signature of RNA polymerase II transcriptional activity, consistent with active enhancer utilization (Figure 1D). Intriguingly, the rs202275 transversion site was found to reside near the site of RNA polymerase II loading within this putative enhancer region (i.e., the bidirectional point of bifurcation), defining a critical functional role for the variant in regulatory function.

To access putative regulation of *FABP5* expression by the rs202275-containing region, we used Micro-C, a high-resolution chromosome conformation capture–based method, to assay physical contacts between the rs202275 region and the *FABP5* transcription start site (TSS) in BEAS-2B airway epithelial cells (NCBI GEO GSE241294). We observed relatively high frequency of physical contact between rs202275 and the *FABP5* TSS (Figure 1E, top) that was consistent with publicly available data in embryonic stem cells (6) (Figure 1E, bottom), suggesting that the SNP region can regulate *FABP5* transcription. To test SNP function in patients, we used previously deposited gene array data (GEO GSE42057) from PBMC samples to show that patients carrying the risk allele (T) of the rs202275 SNP express significantly lower levels of *FABP5* compared with noncarrier patients (Figure 1F). Thus, the data highlight a direct regulation of *FABP5* transcription by the rs202275 variant and other linked SNPs.

To define a functional correlate for regulation of *FABP5* transcription by rs202275, we assessed the metabolic switch from glycolysis to mitochondrial respiration in fresh PBMCs from patients with COPD who carry one or more of the SNP variants. PBMCs were used, as they are the least invasive cells to obtain from patients. As controls, we obtained blood samples from patients with COPD who do not carry the variants (Supplemental Tables 2–4). We previously demonstrated that FABP5 expression is required for the pro- versus antiinflammatory transition in bone marrow–derived macrophages. Real-time mitochondrial respiration was assessed using the Seahorse XF Cell Mito Stress Test. We found decreased maximal respiration and spare capacity in PBMCs from patients carrying the risk allele (T) of rs202275 compared with noncarriers (Figure 1G). These data suggest that rs202275 has a functional impact on PBMC metabolism, potentially promoting systemic inflammation in COPD.

In summary, the linked noncoding SNPs within the *FABP5* locus associated with COPD exacerbations likely exert regulatory functions that inhibit *FABP5* transcription and have downstream effects on cell metabolism, thereby contributing to sustained systemic inflammation during COPD exacerbations.

## Supplementary Material

Supplemental data

Supporting data values

## Figures and Tables

**Figure 1:**
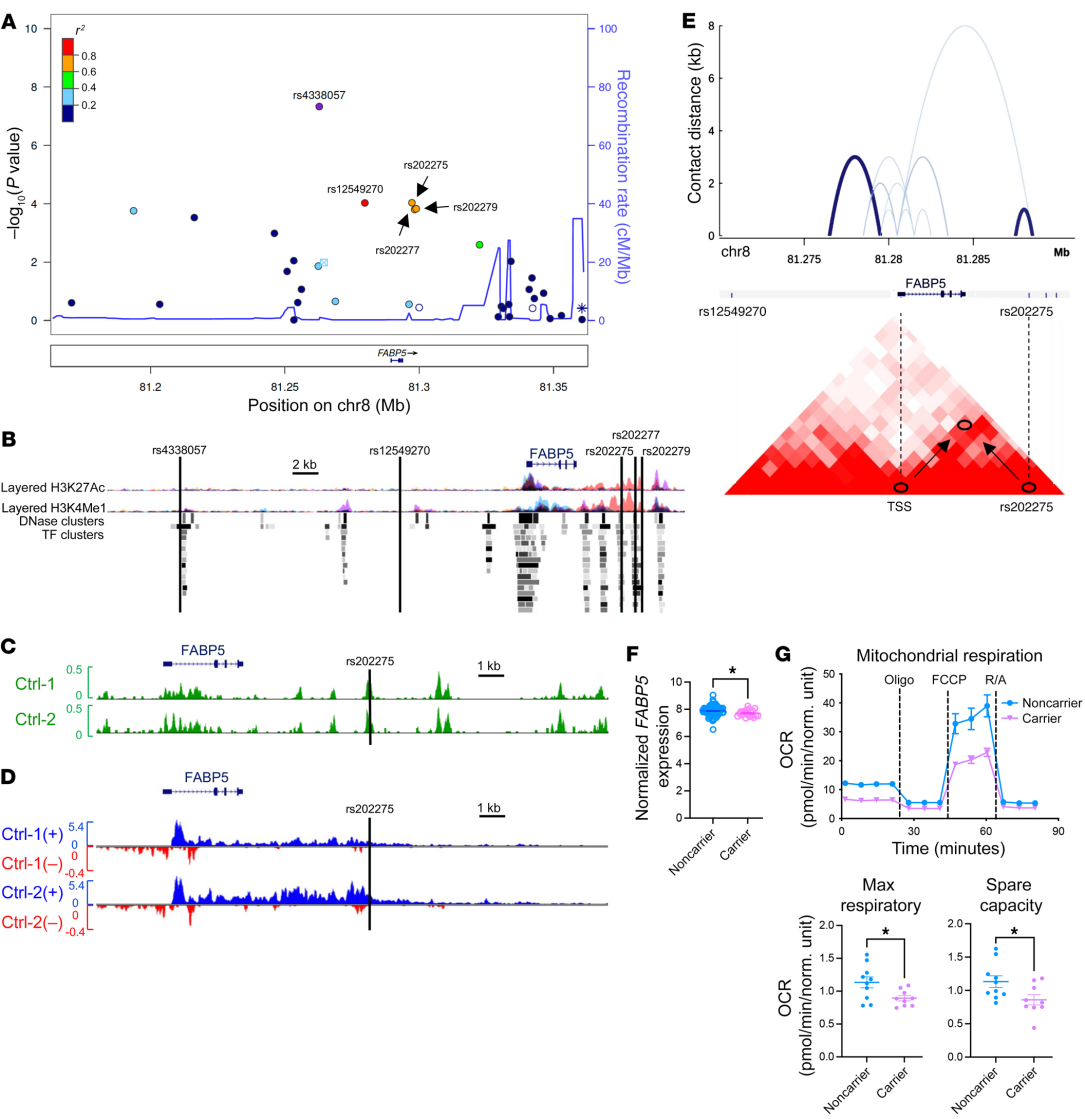
The rs202275 SNP binds regulatory regions of open chromatin and interacts with the *FABP5* transcription start site, decreasing *FABP5* expression and reducing oxidative metabolism in mononuclear cells from COPD patients. (**A**) *FABP5* SNPs associated with prospective severe exacerbations in the COPDGene non-Hispanic White cohort. Data were generated using R (https://www.r-project.org/). *n* = 6,649. (**B**) SNP locations within the *FABP5* locus (black lines) on chromosome 8 (chr8). (**C**) ATAC-seq profiles (reads per million mapped) at the *FABP5* locus showing accessible chromatin at the rs202275 SNP. (**D**) PRO-seq data at the *FABP5* locus showing bidirectionality of the RNA polymerase II loading at rs202275. (**E**) Micro-C interactions at the *FABP5* locus, with arcs (top) and boxes connecting 1-kb interacting regions across genomic space (bottom). TSS, transcription start site. Data in **B**–**E** (bottom) were visualized in the UCSC Genome Browser (https://genome.ucsc.edu/) using airway epithelial cells. (**F**) Normalized gene array expression levels of *FABP5* (GEO GSE42057) in 116 noncarrier and 20 SNP carrier PBMCs. (**G**) Mitochondrial respiration measured by oxygen consumption rate (OCR) in PBMCs by genotype and associated mitochondrial parameters. Data represent 10 COPD noncarrier and 9 COPD SNP carrier donor samples. Data in **F** and **G** represent mean ± SEM. *P < 0.05, groups compared via 2-tailed Student’s *t* test. Experimental details can be found in the Supplemental Methods. All raw data can be found in the supplemental Supporting Data Values file.

## Version 1 12/19/2023

In-Press Preview

## Version 2 02/01/2024

Electronic publication
