# Supplementary material for: Noncoding SNPs decrease expression of FABP5 during COPD exacerbations
Source: J Clin Invest. 2024 Feb 1;134(3):e175626. doi: 10.1172/JCI175626 (PMC10849757; doi:10.1172/JCI175626)
Supplement: Supplemental data [file jci-134-175626-s150.pdf]

SUPPLEMENTARY MATERIALS

for

Noncoding SNPs decrease expression of FABP5 during COPD exacerbations

Contents

METHODS ____ *2*

Assay for transposase-accessible chromatin by sequencing (ATAC-seq) and computational analysis ____ 2

Precision Run-on Sequencing (PRO-seq) and computational analysis ____ 2

Donor samples ____ 4

Genotyping of donors ____ 4

SNP determination ____ 5

Microarray Analysis ____ 6

Metabolic Phenotyping ____ 6

Statistical analysis ____ 6

Study approval ____ 7

Data availability statement ____ 7

AUTHOR CONTRIBUTIONS ____ *8*

ACKNOWLEDGEMENTS ____ *9*

TABLES ____ *10*

Table 1. FABP5 SNPs associated with severe exacerbations in COPDGene non-Hispanic White cohort ____ 10

Table 2. Donor characteristics and SNP status. ____ 10

Table 3. Summary of subject exclusions ____ 11

Table 4. Summary of SNP marker exclusions ____ 11

SUPPLEMENTARY REFERENCES ____ *12*

METHODS

Assay for transposase-accessible chromatin by sequencing (ATAC-seq) and computational analysis

Primary airway epithelial cells cultured at air-liquid interface (ALI) were washed twice with 1X PBS and collected by scraping prior to counting. Approximately 50,000 cells were pelleted and processed in duplicate for Omni-ATAC-seq as described previously (1). Uniquely indexed libraries were pooled and sequenced on an Illumina NextSeq instrument using 75 bp single-end reads (Norm-1) or 37 bp paired-end reads (all other samples) by the BioFrontiers Sequencing Facility at the University of Colorado-Boulder. ATAC-seq data were processed using a standardized Nextflow pipeline (https://github.com/Dowell-Lab/ChIP-Flow). Normalized TDF coverage files (reads per million mapped) output by the pipeline were visualized using IGV.

Precision Run-on Sequencing (PRO-seq) and computational analysis

PRO-seq was performed on small airway epithelial cells as previously described (2). Uniquely indexed libraries were pooled and sequenced on an Illumina NextSeq instrument using 75 bp single-end reads by the BioFrontiers Sequencing Facility at the University of Colorado-Boulder. PRO-seq data were processed using a standardized Nextflow pipeline (https://github.com/Dowell-Lab/Nascent-Flow). A complete pipeline report detailing all software programs and versions utilized and a detailed quality control report including trimming, mapping, coverage, and complexity metrics were previously reported in (3).

Micro-C and computational analysis

Cells were fixed with 1% formaldehyde at room temperature for 10 min and quenched with 0.75 M Tris buffer (pH 7.5). Cells were washed twice with PBS before the addition of 3 mM disuccinimidyl glutarate (DSG) for 45 min at room temperature and quenched with 0.75 M Tris buffer (pH 7.5). Cells were washed twice with PBS, scraped, spun at 1,000 g for 5 min and pellets were snap frozen and stored at -80ºC. Cell pellets were resuspended in 50 mM NaCl, 10 mM Tris-HCl (pH 7.5), 5 mM $MgCl_2$, 1 mM $CaCl_2$, 0.2% NP-40 and 1X protease inhibitor cocktail for 20 min

on ice. Chromatin was digested with micrococcal nuclease for 20 min at 37ºC with shaking at 850 rpm to yield 20% mononucleosomes. The reaction was stopped by the addition of 4 mM EGTA and incubated at 65°C for 10 min. Digested chromatin was washed twice in 50 mM NaCl, 10 mM Tris-HCl (pH 7.5), 10 mM $MgCl_2$. The chromatin was then subjected to T4 Polynucleotide Kinase in 50 mM NaCl, 10 mM Tris-HCl (pH 7.5), 10 mM MgCl2, 100 µg/mL BSA, 2 mM ATP and 5 mM DTT at 37ºC for 15 min prior to the addition of Klenow Fragment at 37ºC for 15 min to generate blunt ends. Labeling was performed by the addition of biotin-dATP, biotin-dCTP, dTTP and dGTP at 66 mM each and incubating at 25ºC for 45 min. Enzymes were inhibited by the addition of 30 mM EDTA and incubation at 65ºC for 20 min. Chromatin was washed once in 50 mM Tris-HCl (pH 7.5) and 10 mM $MgCl_2$. DNA ends between crosslinked nucleosomes were ligated using T4 DNA ligase for 2.5 hours at room temperature. Biotin-dNTPs on the unligated ends were removed by exonuclease III at 37ºC for 15 min. The samples were reverse crosslinked by overnight incubation at 65ºC with 2 mg/mL Proteinase K and 1% SDS. DNA was purified using phenol:chloroform:isoamyl Alcohol twice, run on a 3% TBE NuSieve agarose gel and di-nucleotide bands were extracted and purified using streptavidin beads. Standard library preparation protocol including end-repair, A-tailing and adapter ligation was performed using the NEBnext Ultra II kit. The sequencing library was amplified by Kapa HiFi PCR enzyme with the lowest possible number of cycles to reduce PCR duplicates. MicroC experiments were mapped to hg38 and downstream contact sites were identified using the package HiC-Pro (v2.11.4), which has dependencies of samtools (v1.1) and python (v2.7) and the python packages bx-python (v0.8.9), scipy (v1.2.3), pysam (v0.16.0.1), pandas (v0.24.2), and the iced normalization package (v0.5.7). The configuration for HiC-Pro specified to remove all singleton, multimapped, and duplicate reads. Iced-normalized contact counts output by HiC-Pro were visualized using the plotgardener library (v1.4.2) in R v4.2.3.

Donor samples

Blood samples from the COPDGene cohort were used in this study as previously described (4). COPDGene (www.COPDGene.org) is a National Heart Lung and Blood Institute-funded multicenter observational study designed to identify the genetic risk factors associated with COPD. Cases are diagnosed by post-bronchodilator spirometry as GOLD Stage II or greater (FEV1 < 80% predicted and FEV1/FVC < 0.7). COPD cases are between ages 45 and 80 and have at least a 10 pack-years of smoking history, and can be current or former smokers (5). The follow up study includes blood sample collection. Peripheral blood mononuclear cells (PBMCs) from 9 SNP carriers and 10 non-carriers were freshly isolated using CPT tubes (BD Biosciences, Vacutainer CPT tubes) and separated by density gradient centrifugation (**Supplemental Table 2**). After washing in HBSS, PBMCs were frozen and kept in liquid nitrogen until used to perform FABP5 mRNA quantification and metabolic assays.

Genotyping of donors

Genotyping quality control was performed following previously described guidelines (6, 7). 10,503 DNA samples from COPDGene subjects, including duplicates and controls, were genotyped by Illumina (San Diego, CA) on the HumanOmniExpress array. GenomeStudio quality control, including manual review of cluster plots, was performed by Illumina. Genotype calls and intensities were exported for further quality control. Subjects were assessed for SNP missingness (>1.5%), SNP heterozygosity (6 standard deviations above the mean), chromosomal aberrations (analysis of B allele frequency), gender (X and Y chromosome intensity), and cryptic relatedness by estimated IBD (> 0.125). After genotype cleaning, an additional set of subjects were excluded based for ineligibility in the primary study, including the presence of other lung disease and inadequate smoking history. An additional ten subjects were dropped because of discordance in alpha-1 genotyping and plasma protein phenotyping results.

Principal component analysis was performed to identify racial mismatches and population outliers. Autosomal SNPs present in the HapMap3 dataset with a minor allele frequency of > 5% and

Hardy-Weinberg *p*-value > 0.01 were pruned in plink (8) using an initial r^2 of 0.12 across a 1500 SNP window, and further pruned using an r^2 of 0.05 within a 50 SNP window. Principal components were generated using EIGENSOFT 3.0 (9) and were assessed using both COPDGene and HapMap3 subjects. After removal of racial mismatches, 42 non-Hispanic whites were found to fall beyond six standard deviations on the first three principal components and were removed. No outliers were found in the African American subjects. A summary of the subject exclusions is shown in **Supplemental Table 3**.

Markers were cleaned based on SNP concordance (<99%), missingness (>2% for allele frequency < 5%, otherwise >5%), and Hardy-Weinberg equilibrium in controls ($<10^{-8}$). Markers with low minor allele frequency (<1%) were additionally excluded for the primary analysis. A summary of the excluded markers is shown in **Supplemental Table 4**.

SNP determination

Analyses were conducted using R. Severe exacerbations during longitudinal follow-up, defined as a COPD-related hospitalization or emergency department visit, were modeled with a negative binomial regression with offset for exposure time and a zero-inflation model to account for the excess number of subjects who reported no acute episodes of respiratory disease. Associations of exacerbations to SNPs were first determined without covariates. This model was applied to the subjects in the COPDGene study population with the clinical covariates of age, sex, percent-predicted FEV1, self-report of gastroesophageal reflux, St George's Respiratory Questionnaire (SGRQ), smoking status, and history of prior exacerbation. The zero-inflation component of the model included the clinical covariate percent-predicted FEV1 only. Clinical covariates were selected after reviewing the current literature on exacerbations; only clinical covariates that were significant in several cohort studies were used.

For each SNP, the model fitting returns an estimate, standard error, and *p*-value for the association of the SNP within the count model (negative binomial) and an estimate for the association of the SNP for zero counts within the zero-inflated component of the model (gamma

parameter). The *p*-values were adjusted for multiple testing using the Benjamini-Hochberg method and false discovery rate (FDR) values > 0.05 were considered significant (**Supplemental Table 1**).

Microarray Analysis

We accessed Gene Array data available in the NCBI Gene Expression Omnibus (GEO Accession #GSE42057) from a study that measured the expression of 54,675 transcripts using Affymetrix Human Genome U133 plus 2.0 Gene Array (Affymetrix, Santa Clara, CA) (10). We specifically looked for *FABP5* expression and segregated the data between SNP carriers (SNP+) and non-carriers (SNP-).

Metabolic Phenotyping

Oxygen consumption rate (OCR) and extracellular acidification rate (ECAR) were measured using the Agilent Seahorse XFe96 Bioanalyzer. PBMCs ($20x10^4$ per well) were plated in quadruplets onto Seahorse 96-well plates and pre-incubated in the indicated Seahorse XF RPMI media at 37ºC for 1 hr in the absence of CO_2. OCR was measured under basal conditions and after sequential addition of Oligomycin, Carbonyl cyanide 4-(trifluoromethoxy)phenylhydrazone (FCCP) and Rotenone/Antimycin A. ECAR was measured under basal conditions in glucose-deprived media and after sequential addition of Glucose, Oligomycin, and 2-deoxy-D-glucose (2-DG) following manufacturer's instructions. Each measured value was reported on Wave software (Agilent Technologies) and normalized to the number of cells in each well. The cell count per well was determined by fluorescent cell counting using BioTek Cytation 1/5 instrument.

Statistical analysis

Data are expressed as mean ± SEM. Statistical tests were performed using Prism 8 software (GraphPad). Student's *t* test was used to compare two groups. Differences were considered statistically significant when $p < 0.05$.

Study approval

Human studies were approved by National Jewish Institutional Review Board. Written informed consent was received prior to participation.

Data availability statement

The PRO-seq, ATAC-seq and Micro-C data that support the findings of this study have been deposited in NCBI's Gene Expression Omnibus, accession numbers GSE201152, GSE157691, and GSE241294, respectively. All other data are available in the methods and/or supplementary material of this article including supporting data values.

AUTHOR CONTRIBUTIONS

RDD, ANG, RPB, and FG designed the research studies; MEK, SKS, KA and FG conducted the experiments; MEK, SKS, LS, SJ, KA, AG, CG and FG acquired and analyzed the data; SMM, ANG and RPB provided samples and reagents; FG wrote the manuscript; all authors reviewed, edited, and approved the final manuscript.

ACKNOWLEDGEMENTS

This work was supported by NIH NHLBI R01HL141264 (MEK, SKS, XZ, KA, ANG and FG) and by NHLBI U01 HL089897 and U01 HL089856. The COPDGene study (NCT00608764) is also supported by the COPD Foundation through contributions made to an Industry Advisory Committee that has included AstraZeneca, Bayer Pharmaceuticals, Boehringer-Ingelheim, Genentech, GlaxoSmithKline, Novartis, Pfizer, and Sunovion.

TABLES

Table 1. FABP5 SNPs associated with severe exacerbations in COPDGene non-Hispanic White cohort

SNP	*A1*	*MAF*	*B*	*SE*	*p-value*	*FDR*	*Gamma.p*
rs4338057	C	0.10	0.868297987	0.158946658	4.68613E-08	0.000169778	0.00017138
rs12549270	C	0.10	0.598904392	0.153349968	9.40407E-05	0.005495312	0.00440716
rs202275	T	0.11	0.550065609	0.140716375	9.26645E-05	0.005495312	0.00713781
rs202277	A	0.13	0.526361301	0.139325554	0.000158141	0.007848577	0.01186642
rs202279	T	0.12	0.528250639	0.13925014	0.000148516	0.007636632	0.01037477

SNP: single nucleotide polymorphism, A1: risk allele, MAF: minor allele frequency, B: beta value (effect size), SE: standard error, FDR: false discovery rate

Table 2. Donor characteristics and SNP status.

ID	*Gender*	*Race*	*rs4338057*	*rs12549270*	*rs202275*	*rs202277*	*rs202279*
24981Q	Male	Non-Hispanic White	1	1	1	1	1
21807L	Female	Non-Hispanic White	1	1	1	1	1
25369H	Female	Non-Hispanic White	0	0	1	1	1
22172Z	Male	Non-Hispanic White	0	1	1	1	1
18282E	Female	Non-Hispanic White	1	1	1	1	1
21842N	Male	Non-Hispanic White	1	1	1	1	1
22618N	Female	Non-Hispanic White	1	1	1	1	1
18377P	Female	Non-Hispanic White	1	1	1	1	1
18585W	Female	Non-Hispanic White	1	1	1	1	1
24573B	Male	Non-Hispanic White	0	0	0	0	0
19489F	Male	Non-Hispanic White	0	0	0	0	0
23870C	Male	Non-Hispanic White	0	0	0	0	0
23575A	Male	Non-Hispanic White	0	0	0	0	0
21704B	Female	Non-Hispanic White	0	0	0	0	0
23273K	Male	Non-Hispanic White	0	0	0	0	0
24653Z	Male	Non-Hispanic White	0	0	0	0	0
24762E	Male	Non-Hispanic White	0	0	0	0	0
22488A	Male	Non-Hispanic White	0	0	0	0	0
22754V	Male	Non-Hispanic White	0	0	0	0	0

Footnote: 0 absence of mutation, 1 presence of mutation on one allele, 2 presence of mutation on both alleles.

Table 3. Summary of subject exclusions

COPDGene samples with genotypes	***10,503***
Duplicates	-207
Controls	-4
>1.5% missing genotypes	-6
Gender mismatch	-9
Unintended duplicates & Gender mismatch	-4
Unintended duplicates	-37
Estimated IBD > 0.125	-104
Racial mismatches	-11
Ineligible for primary study	-101
Alpha-1 genotype mismatch	-2
Alpha-1 deficiency	-8
Outliers identified among NHW subjects	-40
**COPDGene subjects passing QC**	**9,970**

Table 4. Summary of SNP marker exclusions

	Non-Hispanic White	***African American***
Missingness	5,282	5,034
Concordance <99%	254	254
HWE $p < 10^{-8}$	1,597	2,322
MAF < 0.01	76,290	19,822
**Remaining SNPs**	**646,125**	701,709

SUPPLEMENTARY REFERENCES

1. Corces MR, Trevino AE, Hamilton EG, Greenside PG, Sinnott-Armstrong NA, Vesuna S, et al. An improved ATAC-seq protocol reduces background and enables interrogation of frozen tissues. *Nat Methods.* 2017;14(10):959-62.
2. Gally F, Sasse SK, Kurche JS, Gruca MA, Cardwell JH, Okamoto T, et al. The MUC5B-associated variant rs35705950 resides within an enhancer subject to lineage- and disease-dependent epigenetic remodeling. *JCI Insight.* 2021;6(2).
3. Gupta A, Sasse SK, Berman R, Gruca MA, Dowell RD, Chu HW, et al. Integrated genomics approaches identify transcriptional mediators and epigenetic responses to Afghan desert particulate matter in small airway epithelial cells. *Physiol Genomics.* 2022;54(10):389-401.
4. Rao DM, Phan DT, Choo MJ, Weaver MR, Oberley-Deegan RE, Bowler RP, et al. Impact of fatty acid binding protein 5-deficiency on COPD exacerbations and cigarette smoke-induced inflammatory response to bacterial infection. *Clin Transl Med.* 2019;8(1):7.
5. Regan EA, Hokanson JE, Murphy JR, Make B, Lynch DA, Beaty TH, et al. Genetic epidemiology of COPD (COPDGene) study design. *COPD.* 2010;7(1):32-43.
6. Laurie CC, Doheny KF, Mirel DB, Pugh EW, Bierut LJ, Bhangale T, et al. Quality control and quality assurance in genotypic data for genome-wide association studies. *Genet Epidemiol.*34(6):591-602.
7. Weale ME. Quality control for genome-wide association studies. *Methods Mol Biol.* 2010;628:341-72.
8. Purcell S, Neale B, Todd-Brown K, Thomas L, Ferreira MA, Bender D, et al. PLINK: a tool set for whole-genome association and population-based linkage analyses. *Am J Hum Genet.* 2007;81(3):559-75.
9. Price AL, Patterson NJ, Plenge RM, Weinblatt ME, Shadick NA, and Reich D. Principal components analysis corrects for stratification in genome-wide association studies. *Nat Genet.* 2006;38(8):904-9.
10. Bahr TM, Hughes GJ, Armstrong M, Reisdorph R, Coldren CD, Edwards MG, et al. Peripheral blood mononuclear cell gene expression in chronic obstructive pulmonary disease. *Am J Respir Cell Mol Biol.* 2013;49(2):316-23.
